# Polysiloxane Bonded Silica Aerogel with Enhanced Thermal Insulation and Strength

**DOI:** 10.3390/ma14082046

**Published:** 2021-04-19

**Authors:** Weilin Wang, Zongwei Tong, Ran Li, Dong Su, Huiming Ji

**Affiliations:** Key Laboratory for Advanced Ceramics and Machining Technology of Ministry of Education, School of Materials Science and Engineering, Tianjin University, Tianjin 300072, China; wangweilin@tju.edu.cn (W.W.); tongzongwei@tju.edu.cn (Z.T.); lir184@tju.edu.cn (R.L.)

**Keywords:** silicon-based aerogel, polysiloxane, organic–inorganic composite, coating, thermal insulation properties, mechanical properties

## Abstract

In order to improve the mechanical properties of SiO_2_ aerogels, PHMS/VTES-SiO_2_ composite aerogels (P/V-SiO_2_) were prepared. Using vinyltriethoxysilane (VTES) as a coupling agent, the PHMS/VTES complex was prepared by conducting an addition reaction with polyhydromethylsiloxane (PHMS) and VTES and then reacting it with inorganic silica sol to prepare the organic–inorganic composite aerogels. The PHMS/VTES complex forms a coating structure on the aerogel particles, enhancing the network structure of the composite aerogels. The composite aerogels can maintain the high specific surface area and excellent thermal insulation properties, and they have better mechanical properties. We studied the reaction mechanism during preparation and discussed the effects of the organic components on the structure and properties of the composite aerogels. The composite aerogels we prepared have a thermal conductivity of 0.03773 W·m^−1^·K^−1^ at room temperature and a compressive strength of 1.87 MPa. The compressive strength is several times greater than that of inorganic SiO_2_ aerogels. The organic–inorganic composite aerogels have excellent comprehensive properties, which helps to expand the application fields of silicon-based aerogels.

## 1. Introduction

SiO_2_ aerogels have many advantages, including low thermal conductivity, low density and large specific surface area [1,2,3]. They can be used for thermal insulation [4,5], oil-water separation [6], wastewater treatment [7], sound insulation [8] and catalysts [9]. However, the mechanical properties of SiO_2_ aerogels are poor. The compressive strength of the SiO_2_ aerogels prepared by ambient pressure drying is generally less than 0.6 MPa [10,11]. This is because SiO_2_ aerogels have a three-dimensional network structure composed of nanoparticles. The network can easily collapse under the action of external forces, resulting in the poor mechanical properties of SiO_2_ aerogels [12]. In order to enhance the mechanical properties of SiO_2_ aerogels, the following methods have been proposed: changing precursor [13,14], organic polymer reinforcement [7,15] and fiber reinforcement [16,17]. We focused on two methods: changing precursor and organic polymer reinforcement.

On the one hand, some precursors are used to replace tetraethyl orthosilicate (TEOS) to prepare the silicon-based aerogels. Common precursors are low-molecular-weight silanes, including methyltrimethoxysilane (MTMS) [18] and vinyltriethoxysilane (VTES) [19]. These precursors all have a non-hydrolyzable group (such as methyl and vinyl), which can enhance the elasticity of aerogels and reduce the fragility of aerogels [20]. However, this method decreases the specific surface area of the silicon-based aerogels [21]. Gen et al. used vinyltrimethoxysilane (VTMS) and vinylmethyldimethoxysilane (VMDMS) as precursors to prepare elastic aerogels [22]. Ma et al. used VTES/TEOS and MTES/TEOS as precursors to prepare the silicon-based aerogels. It was found that the aerogels prepared by using VTES/TEOS had better mechanical properties, and the compressive strength could reach 1.45–3.17 MPa; however, the specific surface area was only 365–488 m^2^/g [23]. However, in these studies, they only focused on improving the mechanical properties by changing precursors. The thermal insulation properties of the composite aerogels were not studied.

On the other hand, through the condensation reaction of silica sol and organic polymers, the last are connected with the aerogel network to form polymer-enhanced composite aerogels. Liu et al. used polyurethane to reinforce SiO_2_ aerogels, which increased the compressive strength of the composite aerogels to 4 MPa. However, the specific surface area significantly decreased to only 346 m^2^/g [24]. With NaOH as a catalyst, Lin et al. prepared the composite aerogels using polyhydromethylsiloxane (PHMS) and TEOS. The specific surface area of the composite aerogels was still small, ranging from 284 to 576 m^2^/g [25].

In our study, in order to comprehensively improve the thermal insulation properties and mechanical properties of the silicon-based aerogels, we used the two methods, changing precursor and organic polymer reinforcement, at the same time. The new precursor (VTES) and polymer (PHMS) were used to prepare the PHMS/VTES complex through an addition reaction. Next, the PHMS/VTES-SiO_2_ composite aerogels (P/V-SiO_2_) were prepared by a condensation reaction of the PHMS/VTES complex and inorganic silica sol. The PHMS/VTES complex formed a coating structure on the aerogel particles, achieving chemical bonding between adjacent aerogel particles, which improved the strength of the aerogel network. Therefore, the mechanical properties of the composite aerogels could be improved, while maintaining the high specific surface area and the thermal insulation properties. We analyzed the structure of the composite aerogels and discussed the effects of PHMS and VTES on the structure and properties of the composite aerogels. It provided a new idea for preparing the silicon-based aerogels with high specific surface area, low thermal conductivity and high strength.

## 2. Materials and Methods

### 2.1. Materials

The following materials were sourced as indicated. Polyhydromethylsiloxane (PHMS, 1.55%, calculated by hydrogen) was from Kaihuasantai, Quzhou, China. Platinum divinyltetramethyldisiloxane complex (2%, calculated by platinum) was from Anpin Silicone Materials, Shenzhen, China. Isopropanol (99%) and formamide (99%) were from Fuchen, Tianjin, China. Triethoxyvinylsilane (VTES, 97%), tetraethyl orthosilicate (TEOS, 98%), trimethylsilylchloride (TMCS, 98%), methyltrimethoxysilane (MTMS, 98%), methyltriethoxysilane (MTES, 98%), nitric acid (63%), propylene oxide (99%) and n-hexane (97%) were all from Aladdin, Shanghai, China. All the materials were of analytical grade and used directly, without further treatment of purification.

### 2.2. Experimental Procedure

The preparation of the PHMS/VTES-SiO_2_ composite aerogels (P/V-SiO_2_) was divided into three stages: (1) preparation of the PHMS/VTES complex, (2) preparation of the wet gels and (3) post-processing of the composite aerogels. All reactions were carried out in beakers.

#### 2.2.1. Preparation of the PHMS/VTES Complex

PHMS and VTES underwent an addition reaction at 45 °C with platinum divinyltetramethyldisiloxane complex as the catalyst. The amount of the catalyst was 1 wt.% of PHMS and VTES. The PHMS/VTES complex was obtained in 10 min.

#### 2.2.2. Preparation of the Wet Gels

Firstly, TEOS, isopropanol and deionized water were mixed at the volume ratio of 1.0:1.6:0.5. Nitric acid was added to adjust the pH to 2. The mixture was reacted for 5 h. Then, propylene oxide was added to adjust the pH to 5 to 6, and formamide was added to optimize the network structure of gels. Calculated by volume, the amount of formamide was 0.16 times of TEOS. Next, the inorganic silica sol and organic components were mixed, and then poured into molds. The formulation is shown in Table 1. The wet gels were generated in approximately 12 h, at 40 °C.

#### 2.2.3. Post-Processing of the Composite Aerogels

The post-process of the composite aerogels included aging, alcohol–water exchange, surface modification and ambient pressure drying. First, the wet gels were soaked in isopropanol and aged at 45–60 °C for 48 h. Next, an alcohol–water exchange was performed at room temperature, and the water in the wet gels was replaced with ethanol and isopropanol. The replacement was performed every 8 h, for a total of 6 times. Third, surface modification was performed to replace the hydrophilic groups on the surface of the wet gels with hydrophobic groups to reduce the collapse of pores due to water evaporation during the drying process. The modification process was performed under two conditions, using n-hexane as the solvent: The first time, trimethylchlorosilane was used; the second time, methyltrimethoxysilane and methyltriethoxysilane were used to obtain a better modification effect [11]. The volume ratio of solvent to modifiers was 9:1. The time for each modification was 24 h, and the temperature was 50 °C. Finally, drying at 80 °C for 3–5 h was conducted to obtain the organic–inorganic composite aerogels.

### 2.3. Characterizations

In this study, the morphology of the composite aerogels was studied by scanning electron microscopy (SEM; Hitachi, S-4800, Tokyo, Japan) at an acceleration voltage of 3 kV. Before SEM analysis, the samples underwent the treatment of gold sputtering to enhance conductivity. The chemical composition of the composite aerogels was characterized by Fourier-transform infrared spectroscopy (FTIR; Thermo, Nicolet iS5, Waltham, MA, USA). The pore structure was analyzed by a Surface Area and Pore Size Analyzer (Quantachrome, NOVA 2200e, Boynton Beach, FL, USA) with N_2_ as an adsorbent. The specific surface area was calculated using Brunauer–Emmett–Teller (BET) method, and the pore size distribution was calculated by using the Barrett–Joyner–Halenda (BJH) method. Before the analysis of pore structure, the samples underwent the treatment of degassing at 130 °C, for 15 h. Thermal conductivity was measured by the hot-wire method, using a thermal conductivity measuring instrument (XIATECH, TC3000, Xi’an, China). During the measurement, the heating voltage was 0.9 V, and the sampling time was 2 s. Compressive strength was measured by a digital mechanical strength measurement instrument (KEBAO, DL-15, Dongguan, China) with a load application speed of 0.1 mm/min.

## 3. Results

### 3.1. Microscopic Morphology and Structural Characteristics of the Composite Aerogels

**Figure 1:**
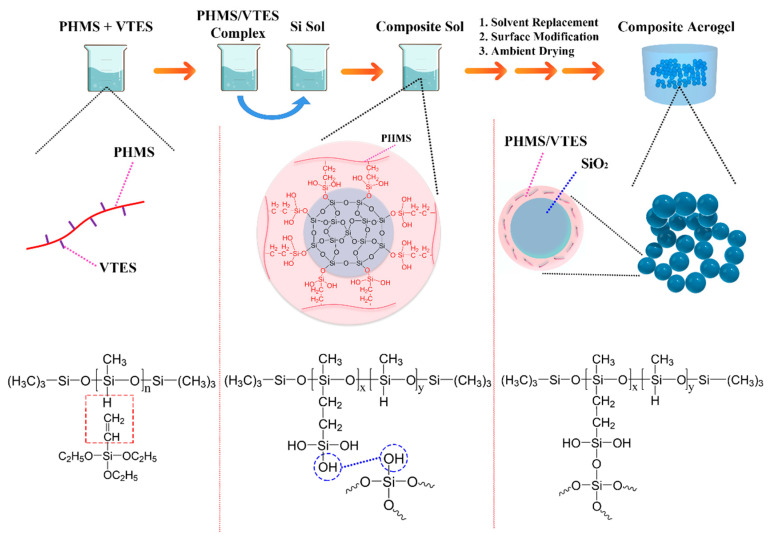
Preparation process and preparation mechanism of the composite aerogels.

The preparation of the composite aerogels can be divided into three stages: (1) The organic polymer (PHMS) and coupling agent (VTES) formed the PHMS/VTES complex by an addition reaction. (2) The PHMS/VTES complex reacted with silica sol to form composite gels. (3) The composite aerogels were obtained from the composite gels by ambient pressure drying. The process and mechanism are shown in Figure 1.

#### 3.1.1. Chemical Composition and Reaction Mechanism

**Figure 2:**
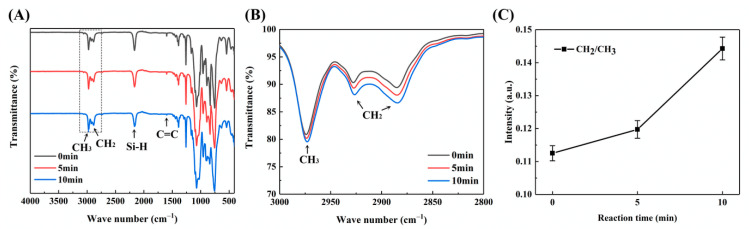
Infrared spectra of the addition reaction of PHMS and VTES: (**A**) complete infrared spectrum; (**B**) enlarged view of part of the spectra shown in (**A**), in the wavenumber range of 2800–3000 cm^−1^; (**C**) quantitative analysis of methylene.

During the addition reaction between PHMS and VTES, the samples in the reaction were analyzed by infrared spectroscopy, as shown in Figure 2A. The peak at 2980 cm^−1^ was attributable to the stretching vibration of methyl group. The peaks at 2926 and 2884 cm^−1^ were attributable to the stretching vibration of methylene group [26]. There were two sources of methylene: the ethoxy (-OCH_2_CH_3_) of VTES and Si-CH_2_-CH_2_-Si generated in the addition reaction between PHMS and VTES. By quantitative analysis of the methylene content, the progress of the addition reaction could be estimated. In quantitative analysis, the stronger absorption peak at 2926 cm^−1^ represented methylene (Figure 2B). The peak intensity was normalized according to the absorption peak of the methyl group at 2980 cm^−1^. As shown in Figure 2C, as the reaction progressed, the stretching vibration peak of methylene increased. This was due to the formation of Si-CH_2_-CH_2_-Si by the addition reaction between Si-H bond and C=C bond, which indicated that the addition reaction proceeded gradually. The absorption peak of C=C bond at 1620 cm^−1^ and the absorption peak of Si-H bond at 2200 cm^−1^ decreased as the reaction progressed, which also proved that the addition reaction was proceeded gradually. After 10 min, the absorption peak of C=C at 1620 cm^−1^ tended to disappear, indicating that PHMS and VTES had sufficiently reacted.

**Figure 3:**
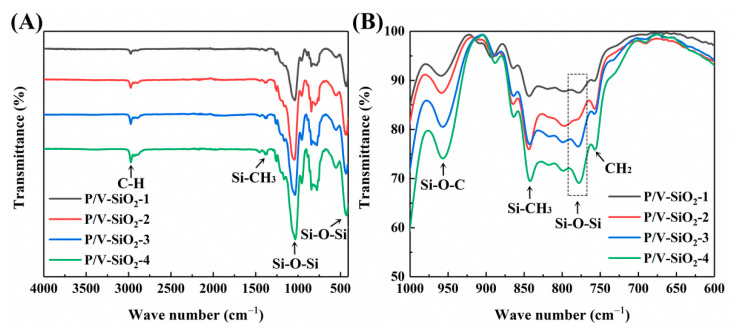
Infrared spectra of the composite aerogels: (**A**) samples P/V-SiO_2_-1, P/V-SiO_2_-2, P/V-SiO_2_-3 and P/V-SiO_2_-4; (**B**) enlarged view of part of the spectra shown in (**A**), in the wavenumber range of 600–1000 cm^−1^.

The infrared spectrum of the composite aerogels is shown in Figure 3A. The absorption peaks of Si-O-Si at 1030, 778 and 442 cm^−1^ are derived from the network of the aerogels and the molecular chain of PHMS [27]. The peak at 1030 cm^−1^ is the asymmetric stretching vibration absorption peak of Si-O-Si, 778 cm^−1^ is attributable to the symmetric stretching vibration absorption peak of Si-O-Si and 442 cm^−1^ is attributable to the bending vibration absorption peak of Si-O-Si. The absorption peaks of Si-CH_3_ are at 1270 and 840 cm^−1^ [28,29]. The Si-CH_3_ comes from PHMS and the modifiers trimethylchlorosilane, methyltrimethoxysilane and methyltriethoxysilane introduced in the surface modification. At 960 cm^−1^, there is a symmetrical stretching vibration absorption peak of Si-O-C [30]. Si-O-C comes from the modifiers methyltrimethoxysilane and methyltriethoxysilane. The in-plane rocking absorption peak of CH_2_ is at 735 cm^−1^ [29], which comes from the CH_2_-CH_2_ group formed in the addition reaction. No absorption peak of the Si-H bond is observed at 2160 cm^−1^, which is due to the reaction between PHMS and formamide in isopropanol solvent, causing the Si-H bond to be consumed. As shown in Figure 3B, with the increase in TEOS, the symmetric stretching vibration absorption peak of Si-O-Si at 778 cm^−1^ increases gradually, which indicates that there are more TEOSs undergoing condensation reaction with VTES. This reaction can improve the symmetry of Si-O-Si network and enhance the absorption peak.

#### 3.1.2. Microscopic Morphology

**Figure 4:**
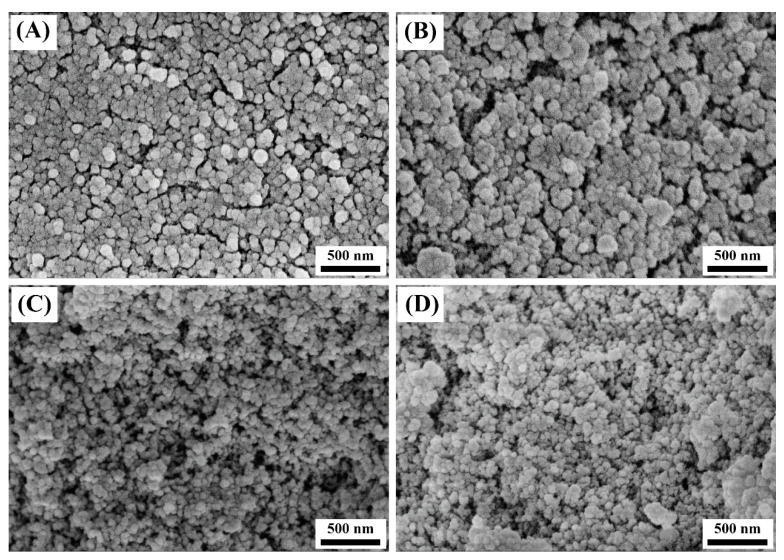
The microscopic morphology of the composite aerogels: (**A**) P/V-SiO_2_-1, (**B**) P/V-SiO_2_-2, (**C**) P/V-SiO_2_-3 and (**D**) P/V-SiO_2_-4.

The microscopic morphology of the composite aerogels is shown in Figure 4. With the decrease in PHMS and VTES, the aerogel particles gradually become smaller, and the particle size decreases from the range of 80–100 nm (the sample P/V-SiO_2_-1 is 85–100 nm, and the sample P/V-SiO_2_-2 is 80–95 nm) to 30–50 nm (the sample P/V-SiO_2_-3 and the sample P/V-SiO_2_-4 are both 30–50 nm). This shows that, when the proportion of PHMS and VTES is high, the coating formed by PHMS and VTES wraps the inorganic aerogel particles, making the aerogel particles large. At this time, the agglomeration phenomenon of the aerogel particles becomes clear (Figure 4A). This is because the PHMS/VTES complex coated on aerogel particles continues to react with the adjacent aerogel particles, resulting in chemical bonding between aerogel particles. It causes the agglomeration of aerogel particles. Therefore, the proportion of PHMS and VTES determines the thickness of the coating, which affects the morphology and particle size of the composite aerogels.

#### 3.1.3. Pore Structure

**Figure 5:**
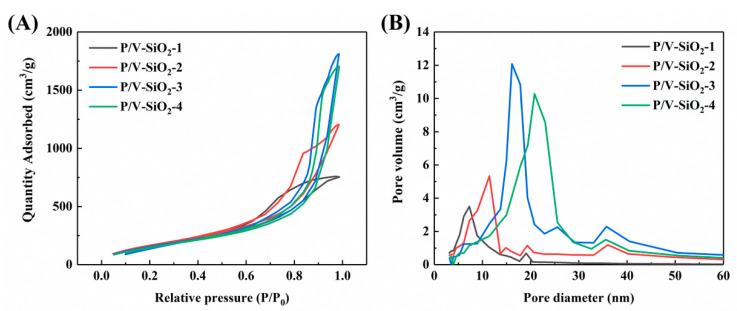
N_2_ adsorption and desorption analysis of the composite aerogels (samples P/V-SiO_2_-1, P/V-SiO_2_-2, P/V-SiO_2_-3 and P/V-SiO_2_-4): (**A**) adsorption and desorption curves and (**B**) pore size distribution.

The N_2_ adsorption and desorption curves of the composite aerogels are shown in Figure 5A. The adsorption and desorption curves are all type IV curves according to the IUPAC classification [31,32], indicating that the composite aerogels are typical mesoporous materials. There is a hysteresis loop in the adsorption and desorption curves that is due to capillary condensation in the mesoporous materials [13]. The sample P/V-SiO_2_-1 shows a type H2(b) hysteresis loop, and the samples P/V-SiO_2_-2, P/V-SiO_2_-3 and P/V-SiO_2_-4 show a type H1 hysteresis loop [33]. These two types of hysteresis loops both show that the pore size distribution is uniform. The pore size distribution of the composite aerogels is shown in Figure 5B. The pore size is mainly in the range of 5–40 nm, which is consistent with the characteristics of mesoporous materials [13]. With the increase in the PHMS/VTES complex, the pore size gradually decreases.

The analysis results of the N_2_ adsorption and desorption of the composite aerogels are shown in Table 2. The most probable pore sizes of the composite aerogels are between 7 and 20 nm. As the proportion of PHMS/VTES increases, the most probable pore size of the composite aerogels decreases. This is because the PHMS/VTES complex can bond with the inorganic Si-O-Si network. A coating structure forms on the aerogel particles, which makes the aerogel particles larger and the pore size smaller. This is consistent with the morphological characteristics shown in Figure 4. The analysis results show that the composite aerogels have high specific surface area. When the pore volume does not significantly change, the smaller the pore size, the larger the specific surface area. Therefore, when the proportion of PHMS/VTES increases, the pore size decreases and the specific surface area of the composite aerogels increases (sample P/V-SiO_2_-2). However, when the size of the aerogel particles further increases, the pore volume of aerogels decreases obviously, thus making the specific surface area of the composite aerogels decrease (sample P/V-SiO_2_-1).

Compared with the typical inorganic SiO_2_ aerogels prepared with ethanol as the solvent, the composite aerogels we prepared have a larger pore size when the proportion of PHMS/VTES is low. This is related to changing in solvent from ethanol to isopropanol [34]. However, the use of isopropanol as a solvent is a necessary condition to achieve the mixing of the organic and inorganic components. It is difficult to achieve similar effects with ethanol.

### 3.2. Properties of the Composite Aerogels

#### 3.2.1. Thermal Properties

The heat-transfer process of aerogels can be divided into two aspects: solid-phase heat transfer and gas-phase heat transfer. The solid-phase heat transfer depends on the aerogel framework, and the gas-phase heat transfer is closely related to the pore structure of aerogels. When the pore size of the aerogel is smaller than the mean free path of air molecules, the gas-phase heat transfer can be significantly reduced, and the thermal insulation properties of aerogels can be improved [35].

**Figure 6:**
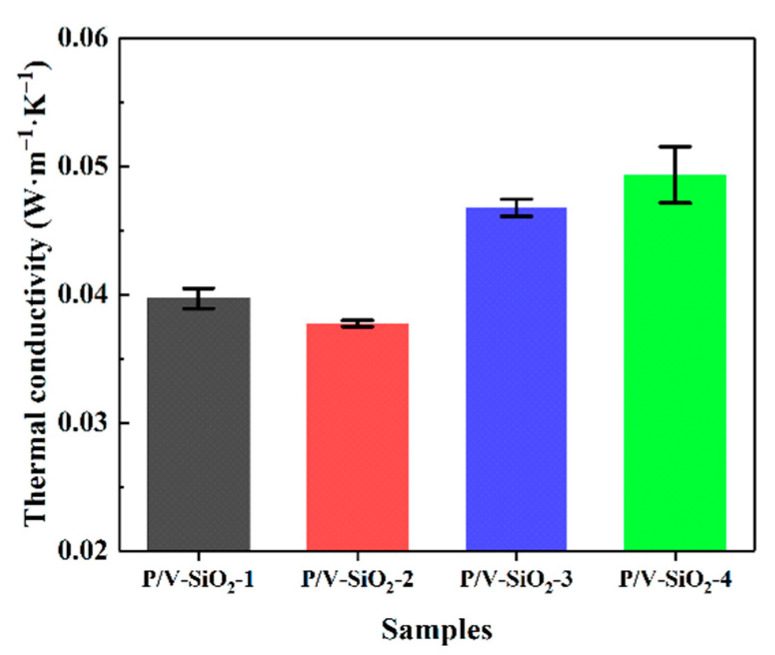
Thermal conductivity of the composite aerogels (samples P/V-SiO_2_-1, P/V-SiO_2_-2, P/V-SiO_2_-3 and P/V-SiO_2_-4).

The thermal conductivity of the composite aerogels is shown in Figure 6. With the increase in PHMS and VTES, the thermal conductivity of the composite aerogels first decreases and then increases. The PHMS/VTES complex forms a coating on the aerogel particles. With the increase in PHMS and VTES, the thickness of the coating is increased (Figure 4). On the one hand, the thickening of the coating helps to improve the specific surface area of the composite aerogels (Table 2), reducing the gas-phase heat transfer and reducing the thermal conductivity of the composite aerogels. On the other hand, when the coating is too thick, the solid-phase heat transfer of the composite aerogels will increase and the thermal conductivity will also increase. Therefore, there is an optimal proportion of PHMS and VTES.

As shown in Figure 6, when the raw-material ratio of the composite aerogels is PHMS:VTES:TEOS=1:0.3:5 (sample P/V-SiO_2_-2), the best heat insulation properties are achieved. Thermal conductivity is as low as 0.03773 W·m^−1^·K^−1^. Under the same conditions, we prepared inorganic SiO_2_ aerogels with only TEOS as the raw material, and its thermal conductivity is 0.03424 W·m^−1^·K^−1^. Compared with the inorganic SiO_2_ aerogels, the thermal conductivity of the composite aerogels has not significantly increased [36]. It is shown that the method of simultaneously introducing long-chain molecules and small-molecule silane to prepare the composite aerogels does not significantly decrease the thermal insulation properties of aerogels.

#### 3.2.2. Mechanical Properties

**Figure 7:**
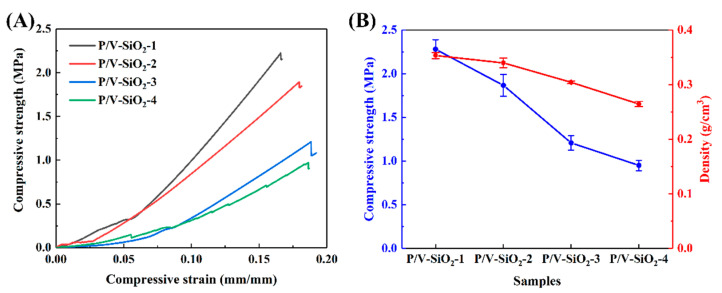
Compressive performance of the composite aerogels (samples P/V-SiO_2_-1, P/V-SiO_2_-2, P/V-SiO_2_-3 and P/V-SiO_2_-4): (**A**) stress–strain curves and (**B**) compressive strength and density.

The stress–strain curves during compression and the compressive properties of the composite aerogels are shown in Figure 7. The compressive strength of the composite aerogels increases with the increase in the proportion of the organic components, PHMS and VTES. In our study, the compressive strength of the composite aerogels was 1.87–2.28 MPa (samples P/V-SiO_2_-1, P/V-SiO_2_-2), while the compressive strength of the inorganic SiO_2_ aerogels was usually less than 0.6 MPa [10,11]. At the same time, we have tested the inorganic SiO_2_ aerogels prepared with isopropanol as a solvent under the same conditions, and the compressive strength was also only 0.740 MPa. Therefore, the mechanical properties of the composite aerogels with the PHMS/VTES complex are significantly better than those of the inorganic SiO_2_ aerogels.

According to Figure 7A, when the proportion of PHMS/VTES is small (samples P/V-SiO_2_-3, P/V-SiO_2_-4), the stress–strain curves of the composite aerogels show the characteristics of typical brittle materials [37]. When the proportion of PHMS/VTES is high (samples P/V-SiO_2_-1 and P/V-SiO_2_-2), the composite aerogels have a large compression shape in the initial stage of the load application. At this stage, the PHMS/VTES coating is the main reason for the deformation. Figure 7B also shows the density of the composite aerogels. With the increase in PHMS and VTES, the density of the composite aerogels gradually increases. This is because the PHMS/VTES complex is coated on the inorganic aerogel particles, resulting in a higher density. The density of the composite aerogels has a positive correlation with mechanical properties, and both of them increase with the increase of PHMS/VTES. Moreover, the PHMS/VTES complex is not only the factor that increases the density of the aerogels but also the factor that enhances the mechanical properties of the aerogels.

## 4. Discussion

We use linear polymer (PHMS) and low-molecular-weight silane (VTES) to form a coating structure on the aerogel particles. The coating affects the structure of the composite aerogels. It is the reason that the composite aerogels have high specific surface area, excellent thermal insulation properties and mechanical properties. In order to obtain this coating structure, we improved the one-pot synthesis method. Firstly, the liquid PHMS/VTES complex was prepared by the addition reaction between PHMS and VTES. Next, the liquid PHMS/VTES complex was mixed with the inorganic silica sol, and the composite aerogels were prepared by the one-pot synthesis method. In this way, the carbon–carbon double bond of VTES avoids being destroyed by strong acid added during the hydrolysis of silica sol, which can ensure the formation of the PHMS/VTES complex.

**Figure 8:**
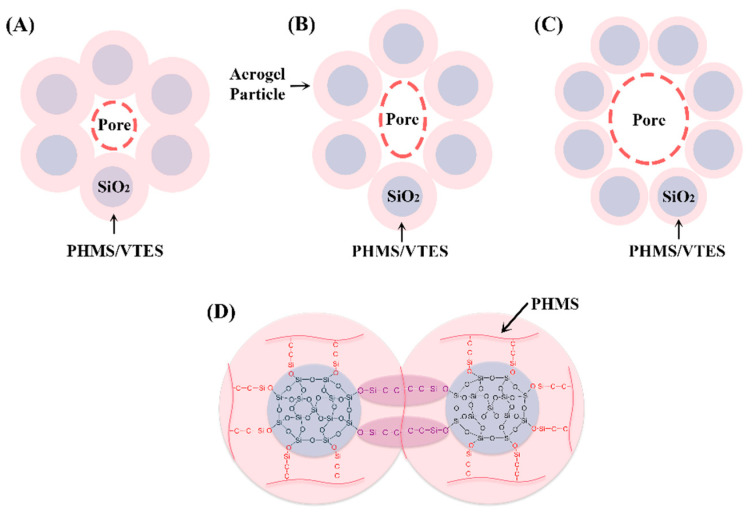
The schematic diagram of the composite aerogel particles with different proportion of PHMS/VTES: (**A**) sample P/V-SiO_2_-1, (**B**) sample P/V-SiO_2_-2 and (**C**) sample P/V-SiO_2_-3. (**D**) The connection method of the adjacent aerogel particles.

Figure 8A–C shows the effect of different proportions of PHMS/VTES on the morphology of the aerogel particles. Firstly, the PHMS/VTES complex forms a coating on the aerogel particles that makes the aerogel particles larger. The organic components have a higher density. With an increase in the particle size, the density of the composite aerogels increase. Secondly, when the proportion of PHMS/VTES is higher, the PHMS/VTES complex accumulated between the aerogel particles is increased, as shown in Figure 8A, which results in the agglomeration of the aerogel particles. The SEM images in Figure 4 prove this. At the same time, the PHMS/VTES complex does not change the porous structure of the aerogels. The composite aerogels can maintain a high specific surface area of more than 600 m^2^/g (Table 2). Therefore, the preparation method we proposed can maintain the advantages of the high specific surface area of the aerogels.

Figure 8A–C shows the two modes of heat conduction of the aerogels: solid-phase heat transfer by the aerogel particles, and gas-phase heat transfer by pores. When the proportion of PHMS/VTES is too high (Figure 8A), the PHMS/VTES coating is too thick, and the particle size and density increase, resulting in an increase in solid-phase heat transfer [38]. When the proportion of PHMS/VTES is too small (Figure 8C), the aerogels have a larger pore size, which makes porosity increase and density decrease, resulting in an increase in gas-phase heat conduction [39]. Both of these conditions cause the thermal conductivity of the aerogels to increase. Therefore, there is an optimal proportion of PHMS/VTES, at which the negative impact of PHMS/VTES on the thermal insulation properties of the composite aerogels is minimized (Figure 6). This is the reason that sample P/V-SiO_2_-2 exhibits the best thermal insulation properties.

Figure 8D shows the chemical bond between the composite aerogel particles. Due to the PHMS/VTES complex, chemical bonds can form between the aerogel particles. Therefore, the adjacent aerogel particles are connected by the PHMS/VTES complex. As the proportion of PHMS/VTES increases, the agglomeration of the aerogel particles becomes clear (Figure 4). The connection strength of the secondary particles is improved [40], which enhances the compressive strength of the composite aerogels (Figure 7).

Considering the thermal insulation properties (Figure 6) and the mechanical properties (Figure 7), the sample P/V-SiO_2_-2 has the best thermal insulation properties and better mechanical properties, achieving a balance between thermal insulation properties and mechanical properties. It proves that the composite aerogels we prepared can achieve an improvement in comprehensive properties.

## 5. Conclusions

In this study, we prepared the PHMS/VTES-SiO_2_ composite aerogels with low thermal conductivity and high compressive strength. It is proved that the PHMS/VTES complex can be compounded with SiO_2_ aerogel particles by wrapping. Through this compound method, the mechanical properties of aerogels can be improved. At the same time, the network structure of aerogels is maintained, and the thermal insulation properties is not significantly reduced. We discuss the effect of the PHMS/VTES complex on the network structure of the composite aerogels, thermal insulation properties and mechanical properties. When the composite aerogel ratio of PHMS:VTES:TEOS is 1:0.3:5, the best thermal insulation properties and better mechanical properties are obtained. At this ratio, the thermal conductivity is 0.03773 W·m^−1^·K^−1^ and the compressive strength is 1.87 MPa, which achieves a balance between thermal insulation and mechanical properties. This study expands the research platform for composite aerogels and proposes a combination approach in which long-chain molecules and low-molecular-weight silanes are introduced. This concept should provide a basis for improving the mechanical properties and network stability of silica aerogels.

## Figures and Tables

**Table 1:** Formulation of the organic–inorganic composite aerogels (mole ratio).

Samples	PHMS *	VTES	TEOS
P/V-SiO_2_-1	1.0	0.3	3.5
P/V-SiO_2_-2	1.0	0.3	5.0
P/V-SiO_2_-3	1.0	0.3	6.5
P/V-SiO_2_-4	1.0	0.3	8.0

* The amount of PHMS is calculated based on the hydrogen content of PHMS.

**Table 2:** N_2_ adsorption and desorption analysis results of the composite aerogels.

Samples	BET Specific Surface Area (m^2^/g)	Pore Volume (cm^3^/g)	Pore Size (nm)
P/V-SiO_2_-1	622.8	1.205	7.288
P/V-SiO_2_-2	673.1	1.922	11.43
P/V-SiO_2_-3	631.3	2.903	16.12
P/V-SiO_2_-4	599.7	2.691	20.82

## Data Availability

Data are contained within the article.
